# MYBL2 regulates de novo purine synthesis by transcriptionally activating IMPDH1 in hepatocellular carcinoma cells

**DOI:** 10.1186/s12885-022-10354-4

**Published:** 2022-12-09

**Authors:** Jun-Zhang Zhao, Wei Wang, Tao Liu, Lei Zhang, De-Zheng Lin, Jia-Yin Yao, Xiang Peng, Gang Jin, Tian-Tian Ma, Jin-Bo Gao, Fang Huang, Jun Nie, Qing Lv

**Affiliations:** 1 Department of Gastrointestinal Surgery, Union Hospital, Tongji Medical College, Huazhong University of Science and Technology, 430022 Wuhan, China; 2 Department of Gastroenterology, The Sixth Affiliated Hospital of Sun Yat-sen University, 510655 Guangzhou, China; 3 Department of Pancreatic-hepatobiliary Surgery, The Sixth Affiliated Hospital of Sun Yat-sen University, 510655 Guangzhou, China; 4 Guangdong Provincial Key Laboratory of Colorectal and Pelvic Floor Diseases, Supported by National Key Clinical Discipline, Guangdong Institute of Gastroenterology, 510655 Guangzhou, China; 5 Department of Endoscopic Surgery, The Sixth Affiliated Hospital, Sun Yat-Sen University, 510655 Guangzhou, China; 6 Department of Thoracic Surgery, Union Jiangnan Hospital, Huazhong University of Science and Technology, Hubei 43022 Wuhan, China; 7 Cancer Center, Union Hospital, Tongji Medical College, Huazhong University of Science and Technology, Hubei 43022 Wuhan, China; 8 Department of Thoracic Surgery, Union Hospital, Tongji Medical College, Huazhong University of Science and Technology, Hubei 43022 Wuhan, China

**Keywords:** MYBL2, IMPDH1, Purine synthesis, HCC

## Abstract

**Background:**

Metabolic reprogramming is a hallmark of cancer, alteration of nucleotide metabolism of hepatocellular carcinoma (HCC) is not well-understood. MYBL2 regulates cell cycle progression and hepatocarcinogenesis, its role in metabolic regulation remains elusive.

**Patients and methods:**

Copy number, mRNA and protein level of MYBL2 and IMPDH1 were analyzed in HCC, and correlated with patient survival. Chromatin Immunoprecipitation sequencing (Chip-seq) and Chromatin Immunoprecipitation quantitative polymerase chain reaction (ChIP-qPCR) were used to explore the relationship between MYBL2 and IMPDH1. Metabolomics were used to analyze how MYBL2 affected purine metabolism. The regulating effect of MYBL2 in HCC was further validated in vivo using xenograft models.

**Results:**

The Results showed that copy-number alterations of MYBL2 occur in about 10% of human HCC. Expression of MYBL2, IMPDH1, or combination of both were significantly upregulated and associated with poor prognosis in HCC. Correlation, ChIP-seq and ChIP-qPCR analysis revealed that MYBL2 activates transcription of IMPDH1, while knock-out of MYBL2 retarded IMPDH1 expression and inhibited proliferation of HCC cells. Metabolomic analysis post knocking-out of MYBL2 demonstrated that it was essential in de novo purine synthesis, especially guanine nucleotides. In vivo analysis using xenograft tumors also revealed MYBL2 regulated purine synthesis by regulating IMPDH1, and thus, influencing tumor progression.

**Conclusion:**

MYBL2 is a key regulator of purine synthesis and promotes HCC progression by transcriptionally activating IMPDH1, it could be a potential candidate for targeted therapy for HCC.

**Supplementary Information:**

The online version contains supplementary material available at 10.1186/s12885-022-10354-4.

## Introduction

Liver cancer is the fifth common malignancy and the second leading cause of mortality worldwide. Hepatocellular carcinoma (HCC) accounts for around 90% of liver cancer, the rest are cholangiocarcinoma (CCA) or mixed forms [1]. HCC has a yearly fatality ratio of approximately, indicating that most patients do not survive a year [1, 2]. The poor outcomes are mainly due to high recurrence of HCC after surgery and resistance to chemotherapy [1]. Moreover, incidence and the overall number of deaths due to liver cancer have rapidly increased in the past decades worldwide [2]. Major risk factors of HCC are viral hepatitis, mainly related to hepatitis B virus (HBV) or hepatitis C virus (HCV) infection, hepatotoxins, and alcohol abuse, whereas epidemiology researches indicate nonalcoholic fatty liver disease (NAFLD) also increases HCC incidence [3, 4].

Recently, metabolic reprogramming, especially nucleotide metabolism, is considered an initiating factor of tumorigenesis and tumor progression [5, 6]. Purine nucleotides metabolism is essential for cells, it not only constitute the building blocks of nucleic acids but also play central roles in enzyme cofactors incorporating, represent as energy source for translation and microtubule polymerization, and intermediates of purine metabolism are involved in signal transduction and angiogenesis [7, 8]. To support rapid proliferation, tumor cells must utilize biosynthetic precursors derived from glycolytic and TCA cycle intermediates for purine and pyrimidine nucleotides biosynthesis de novo or via salvage pathways. This process is limited by the nucleotide pool size as well as level and activity of different rate-limiting enzymes of the nucleotide synthetic pathway [9, 10]. In purine biosynthesis, phosphoribosyl pyrophosphate (PRPP) provides the initial substrate for a series of enzymatic reactions leading to generation of adenine and guanine monophosphate [11]. IMP dehydrogenase (IMPDH) catalyzes the oxidative reaction of IMP to xanthosine 5’-monophosphate (XMP) [12]. The reaction catalyzed by the IMPDH is a rate-limiting step in the de novo biosynthesis of purine nucleotides [12]. Previous studies demonstrated that IMPDH was associated with cell growth, malignant transformation and differentiation [13–15]. It has been reported that IMPDH is involved in different types of malignancies, including methotrexate (MTX)-resistant erythroleukemia, colorectal cancer [16–18].

Oncogenic transformed cancer cells are characterized by altered metabolism, including heightened nutrients uptake, enhanced glycolysis, glutaminolysis and fatty acid synthesis [19, 20]. There are few studies focusing on purine nucleotides metabolism [21–23]. Homolog-like2 B-Myb (MYBL2), a member of Myb family, is a transcriptional factor ubiquitously expressed in tissues. MYBL2 induces fast growth and progression of premalignant, malignant liver through cell cycle deregulation and activation of genes related to hepatocarcinogenesis [24, 25]. In other cell lines, MYBL2 paly versatile roles in cell cycle checkpoint control, cellular senescence, aging, autophagy, and regulation of hematopoietic stem cell [24, 26–28]. To our knowledge, there is no evidence showing the relationship between MYBL2 and cancer metabolism.

We systematically investigated genetic alterations, mRNA and protein expression of MYBL2 in HCC human tumors, and explored possible downstream pathways controlled by MYBL2 using bioinformatic tools. Furthermore, using a combination of metabolomics and genomic analysis, we demonstrate that MYBL2 promotes de novo purine anabolism by up-regulating IMPDH1 at transcriptional level, facilitating tumor growth in vivo. These data provided a potential mechanism of HCC growth and progression from the perspective of nucleotide metabolism.

## Materials and methods

### Cell culture

HepG2 cell line was purchased from ATCC. Cells cultured in DMEM supplemented with 10% fetal bovine serum (Hyclone, Logan, USA), 1% penicillin and streptomycin at 37 °C under 5% CO_2_.

### Western blot

Total protein from cultured cells was extracted in RIPA buffer, and quantified with BCA assay. Protein samples were separated by 4–20% SDS-PAGE, transferred to PVDF membranes, and immunoblotted with antibodies against β-actin (Abcam, ab8227), MYBL2 (Abcam, ab191064), IMPDH1 (Abcam, ab33039) and c-Myc (Abcam, ab32072), subsequently with corresponding HRP-conjugated secondary antibodies (Abcam, ab6721). The immunoreactive bands were visualized by chemiluminescence assay.

### CRISPR/Cas9-mediated genome editing

The lentiviral CRISPR MYC or MYBL2 knockout vectors were constructed by cloning of MYC or MYBL2 guide RNA (gRNA) sequences into the site of BsmBI of lentiCRISPRv2 vector as described [29]. LentiCRISPRv2 without gRNA was used as negative control. PsPAX2 and pCMV-VSV-G were used as lentiviral packaging plasmids. According to the manufacturer’s instructions, transfection of psPAX2, pCMV-VSV-G and lentiCRISPRv2 was carried out in 293T cell lines by using lipofectamine LTX Plus reagent to package lentiviruses.

### Liquid chromatography-mass spectrometry

Cells were incubated with fresh media for 2 h before collection. Cells were washed with ice-cold saline, lysed with 80% methanol in water and quickly scraped into an Eppendorf tube followed by three freeze–thaw cycles in liquid nitrogen at the time of collection. The supernatant was obtained and transferred to a new tube after insoluble material was pelleted in a 4 °C centrifuge, then evaporated to dryness using a SpeedVac concentrator (Thermo Savant). In order to move debris, metabolites were reconstituted in 100 µl of 0.03% formic acid in LC-MS-grade water, vortex-mixed and centrifuged. LC-MS/MS was performed by using an AB QTRAP 5500 liquid chromatography/triple quadrupole mass spectrometer (Applied Biosystems SCIEX) as described previously with an injection volume of 20 µL [30]. MultiQuant software version 2.1 (Applied Biosystems SCIEX) was used for chromatogram review and peak area integration. Statistical analyses were performed using MetaboAnalyst 3.0 (http://www.metaboanalyst.ca).

### RT-PCR

Total RNA was extracted by Trizol agent (15596-026, Invitrogen) in accordance with the manufacturer’s instructions. Total RNA samples were reversely transcribed by 5 × All-In-One RT MasterMix (abmGood). GAPDH was used as internal control for quantification of target genes. Real-time PCR was performed with UltraSYBR Mixture (1,725,121, Bio-Rad) on Bio-Rad IQ5. Primers for IMPDH1 (forward: 5ʹ-GCACACTGTGGGCGAT-3ʹ, reverse: 5ʹ-GAGCCACCACCAGTTCA-3ʹ) and GAPDH (forward: 5ʹ-CATCTTCCAGGAGCGAGATC-3ʹ, reverse: 5ʹ-GCTTGACAAAGTGGTCGTTG-3ʹ) were used.

### Mouse studies

The establishment of HCC xenograft model was described previously [31]. All animal procedures were carried out according to the guidelines issued by the committee on animal research of Union Hospital of Tongji Medical College of Huazhong University of Science and Technology as approved by the Human Ethics Review Board. NSG mice were provided by Cyagen Biosciences China. For cell-line xenografts, cultured cells were collected, washed with PBS, resuspended for injection in serum-free RPMI, 1% penicillin/streptomycin (Sigma #P0781), 25% high-protein Matrigel (Corning #354,248) on ice. Subcutaneous injections were performed in the right flank of 6-8-week-old female NSG mice at 0.2-1 × 10^6^ live cells/mouse in a final volume of 100 µl. Subcutaneous tumor size was measured twice a week with calipers until any tumor reached 2 cm in its largest diameter, calculated by 0.5 x short diameter^2^ x long diameter.

### Immunohistochemical staining

Paraffin-embedded tissue sections were deparaffinized in xylene and rehydrated in graded alcohol after fixing with formalin. 3% hydrogen peroxide in methanol was used to block endogenous peroxidase activity for 10 min. Antigen retrieval was carried out by autoclave sterilization in sodium citrate buffer for 3 min. Slides were incubated with 10% normal goat serum solution for 20 min so as to reduce background non-specific staining. The rabbit polyclonal antibody against IMPDH1, MYBL2 was applied at a concentration of 1:100 and incubated at 4 °C overnight. HRP-conjugated secondary antibody was carried out according to the manufacturer’s instructions. The slides were subsequently incubated with DAB to visualize expression of IMPDH1, MYBL2 and followed by hematoxylin counterstaining. Image capture was performed by using a RGB JVC solid-state camera connected to an Olympus BH2 microscope at 10- and 20-fold objective magnification fitted with a motorized stage.

Immunohistochemical analysis was performed in accordance with standard procedures in a blinded fashion by two pathologists independently. The expressions level was accessed semi-quantitatively based on proportion and staining intensity of positive stained cells. Proportion was scored using semiquantitative criterion: 0 (no staining); 1, minimal (< 10%); 2, moderate (10–50%); and 3, diffuse (> 50%) staining cells. Staining intensity was also classified as 0 (negative); +1 (weak); +2 (moderate); and + 3 (strong). Taken two scores, composed of both proportion and staining intensity together, were added to give each case final expression scores as (0), negative; 1+ (1 or 2), weakly positive; 2+ (3 or 4), moderately positive; 3+ (5 or 6), strongly positive.

### Statistical analysis

**Fig. 1:**
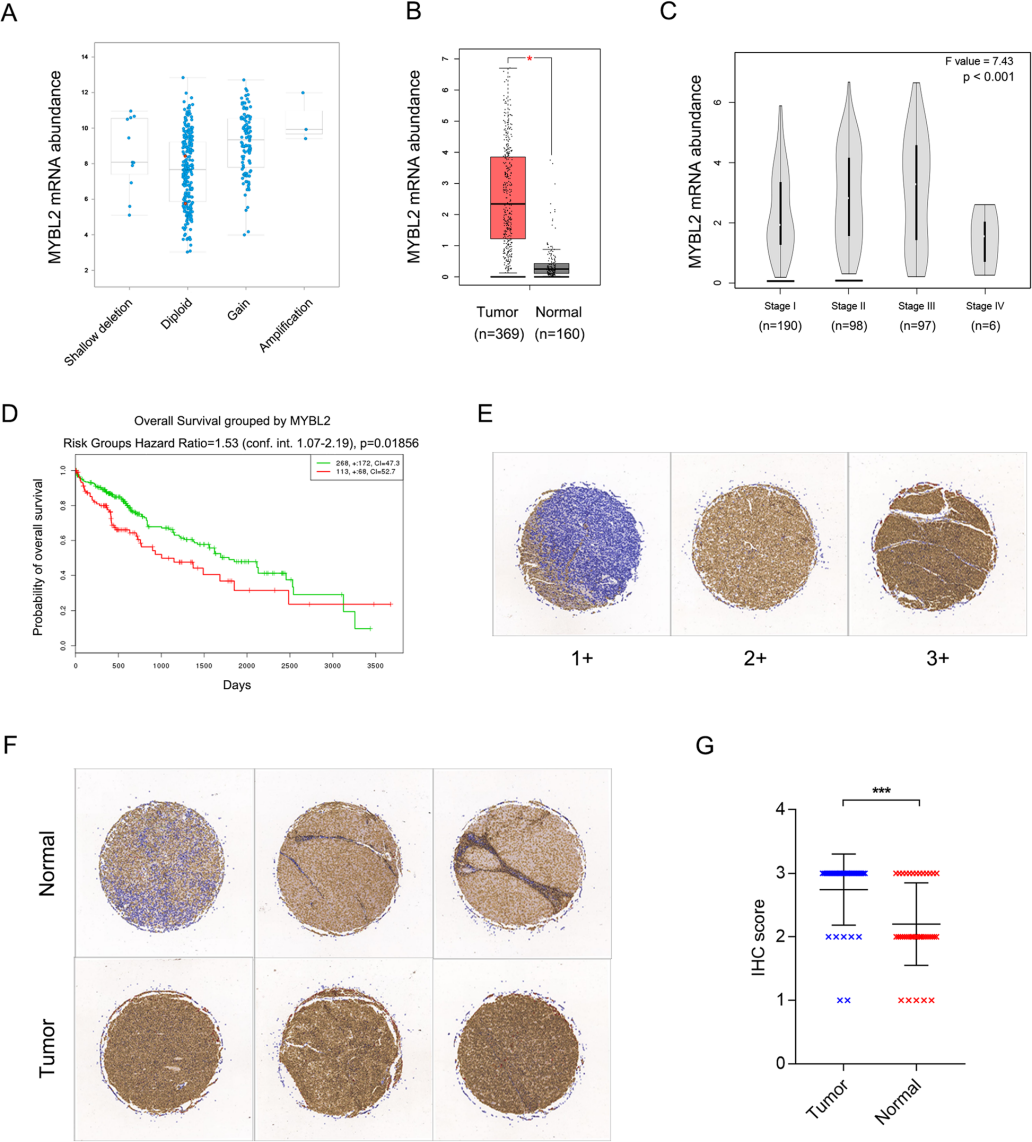
Copy number alterations and expression of MYBL2 in HCC. **A** Copy number alterations of MYBL2 in 360 HCC patients. Data was from TCGA PanCancer Atlas. Gene-level copy number of MYBL2 was generated by GISTIC 2.0 analysis, mRNA expression of MYBL2 was shown as RSEM (Batch normalized from Illumina HiSeq_RNASeqV2) log2(value + 1). **B** mRNA abundance of MYBL2 in 369 HCC patients from TCGA PanCancer Atlas and 160 normal liver tissues from TCGA and GTEx database. **C** mRNA abundance of MYBL2 in HCC patients shown in B stratified by clinical stages. The number of cases at each pathological stage were: Stage I 190, Stage II 98, Stage III 97, Stage IV 6. **D** Overall survival of 381 HCC patients stratified by mRNA abundance of MYBL2. Data was from TCGA database. **E**, **F**. Protein expression of MYBL2 in 35 HCC tumors and matched adjacent normal tissues determined by immunohistochemical (IHC) analysis using tissue microarray. Representative images of different staining intensity and matched tumor and normal tissues were shown. **G**. IHC scores of MYBL2 in HCC tumors and normal adjacent tissues in F. Individual data points are shown with mean and SD. *** *P* < 0.001

Data were shown as means ± S.D. Statistical analysis was performed using a software package (SPSS, version 19.0, Chicago, IL, USA). Analysis of the clinic pathological features and data were carried out with Person’s Chi-Square and Likelihood Ratio tests. A level of *p* < 0.05 was considered significant. Analysis of the relationship between MYBL2 mRNA expression and pathological stages in Fig. 1C was done with one-way ANOVA. Analysis of patient survival was done using Cox Survival method, overall survival data was grouped by mean value of mRNA expression.

## Results

### Copy number alterations and expression of MYBL2 in HCC

Recent studies reported genetic alterations in MYBL2 gene locus, and contributes to tumorigenesis in colorectal, prostate and breast cancer. To understand the role of MYBL2 in HCC, we looked into the liver cancer cohort of the TCGA database. While genetic mutations are rare in MYBL2 gene locus, we found copy number gain in about 10% of HCC tumors, which led to increased mRNA expression (Fig. 1A). Besides genetic alterations, mRNA abundance of MYBL2 were significantly unregulated in HCC tumors compared with normal liver tissues from TCGA normal and GTEx database (Fig. 1B). MYBL2 mRNA expression increased accordingly from clinical stage I to stage III, except for stage IV owing to very few samples collected by surgery resection in TCGA database (Fig. 1C). Notably, higher MYBL2 expression showed worse prognosis of HCC patients (Fig. 1D), suggesting a possible role in HCC tumorigenesis or progression.

To verify the findings of MYBL2 in the liver cancer cohort in TCGA database, which is largely based on DNA and RNA sequencing. We checked protein expression of MYBL2 in 35 HCC tumors and matched adjacent normal tissues by immunohistochemical (IHC) analysis using tissue microarray. Protein abundance were measured by IHC score ranging 1 + to 3+, as shown by representative images of different staining intensity (Fig. 1E). Consistently, we observed a significant higher levels of MYBL2 in HCC tumors compared with adjacent normal liver tissues (Fig. 1F, G).

### MYBL2 mRNA and protein expression correlates with IMPDH1 in HCC

**Fig. 2:**
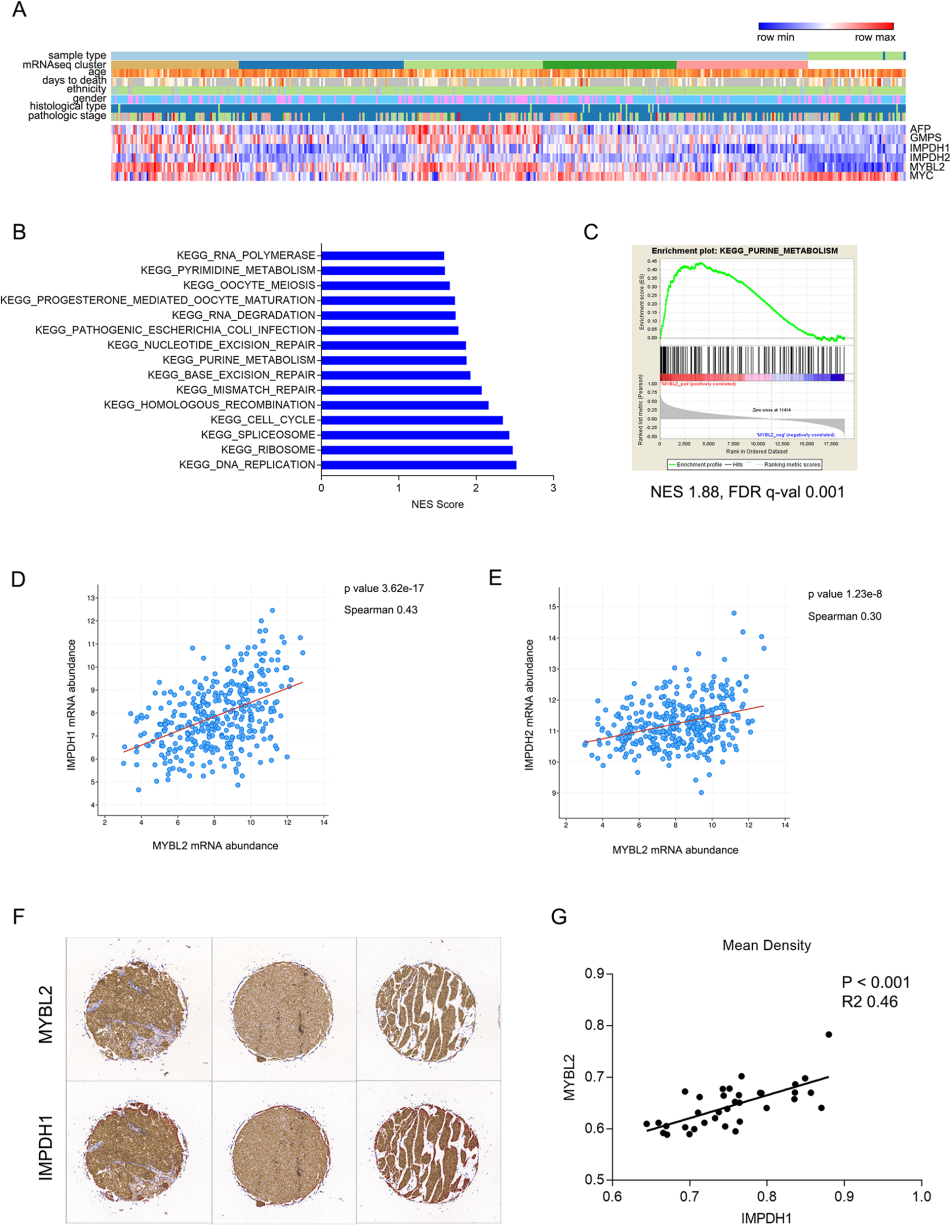
MYBL2 mRNA and protein expression strongly correlates with IMPDH1 in HCC. **A** MYBL2 mRNA abundance correlates with molecular subtypes of HCC patients. mRNA abundance of AFP, GMPS, IMPDH1, IMPDH2, MYBL2 and MYC, and patient clinical data were shown, data was from TCGA database. **B**, **C** KEGG gene signatures [32–34] and purine metabolism gene set correlating with MYBL2 mRNA abundance. Enrichment significance were evaluated with Gene Set Enrichment Analysis (GSEA). **D**, **E** Correlation of IMPDH1 and IMPDH2 mRNA abundance with MYBL2 mRNA abundance, data was from TCGA database. **F** Protein expression of MYBL2 and IMPDH1 in 35 HCC tumors determined by immunohistochemical (IHC) analysis using serial section tissue microarray. Representative images of matched serial tumor sections were shown. **G** Correlation of IMPDH1 protein abundance with MYBL2 protein abundance determined by mean density of IHC staining in **F**

Following investigation of expression profile of MYBL2 in HCC, we subsequently explored functional cues of this potentially important gene. We checked the relationship of MYBL2 expression with clinical and phenotypic parameters, while no correlation with clinical stages and pathological features were seen, MYBL2 aligned well with molecular subtypes of HCC based on unsupervised clustering of transcriptomic data (Fig. 2A). This prompted us to understand whether it drove or helped with certain subtype of HCC. Correlation analysis of the whole transcriptome with MYBL2 mRNA abundance showed multiple signatures were enriched in MYBL2 high tumors, including DNA replication, cell cycle and nucleotide metabolism, especially purine metabolism (Fig. 2B, C).

MYBL2 is a known transcription factor playing pluripotent roles in cell proliferation, cell cycle regulation and cell differentiation. We were interested in the function of MYBL2 in purine metabolism, which is not reported before. Based on our analysis, the metabolic gene most positively correlated with MYBL2 was IMPDH1, in contrast, the functional redundant isoform IMPDH2 was not that positively correlated (Fig. 2D, E). IMPDH is a rate-limiting enzyme in de novo purine synthesis, implying MYBL2 could be a potentially important regulator of purine metabolism. We further validated the correlation between MYBL2 and IMPDH1 protein abundance in serial sections of 35 HCC tumors by IHC analysis using tissue microarray. Interestingly, we observed a significant positive correlation between MYBL2 and IMPDH1 in HCC tumors (Fig. 2F, G).

### IMPDH1 is upregulated in HCC and promotes tumor growth in HCC patient-derived xenograft models

**Fig. 3:**
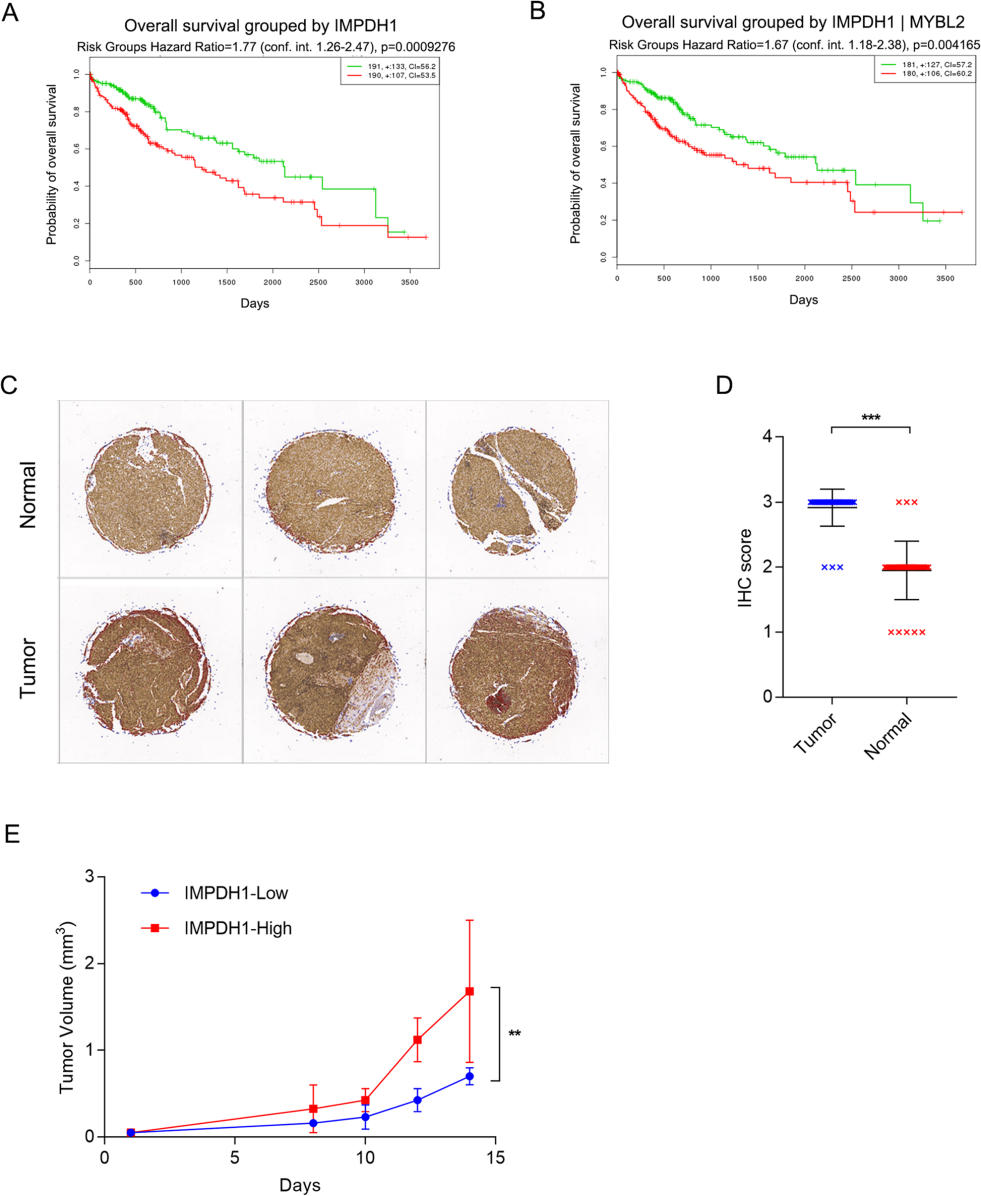
IMPDH1 is upregulated in HCC and promotes tumor growth in HCC patient-derived xenograft models. **A**, **B** Overall survival of 381 HCC patients stratified by mRNA abundance of IMPDH1 with or without MYBL2. Data was from TCGA database. **C** Protein expression of IMPDH1 in 35 HCC tumors and matched adjacent normal tissues determined by immunohistochemical (IHC) analysis using tissue microarray. Representative images of matched tumor and normal tissues were shown. **D** IHC scores of IMPDH1 in HCC tumors and normal adjacent tissues in **C** Individual data points are shown with mean and SD. *** *P* < 0.001. **E** Growth curve of xenografts (*n* = 6 in each group) derived from HCC patients with high or low IMPDH1 levels determined by IHC staining. Individual data points are shown with mean and SD. ** *P* < 0.01

Upregulation of IMPDH is reported in different tumor types. In liver cancer cohort of TCGA database, overall survival analysis showed high mRNA abundance of IMPHD1 represented worse clinical outcome (Fig. 3A). Taken MYBL2 and IMPDH1 together, survival difference was even more obvious, the risk ratio of high to low expression is 2.02 (Fig. 3B). We checked protein expression of IMPDH1 in 35 HCC tumors and matched adjacent normal tissues by IHC analysis using tissue microarray. Protein abundance were measured by IHC score ranging 1 + to 3+, we observed a significant higher levels of IMPDH1 in HCC tumors compared with adjacent normal liver tissues (Fig. 3C, D). To validate the functional importance of IMPDH1 in HCC, we collected small chunks of fresh HCC tumors from patients with low or high expression of IMPDH1, and implanted in the flank of nude mice to generate xenografts. Tumors with high levels of IMPDH1 showed a faster tumor formation and growth compared with tumors with low levels of IMPDH1 (Fig. 3E).

### MYBL2 reprograms purine metabolism and is required for HCC tumor growth

**Fig. 4:**
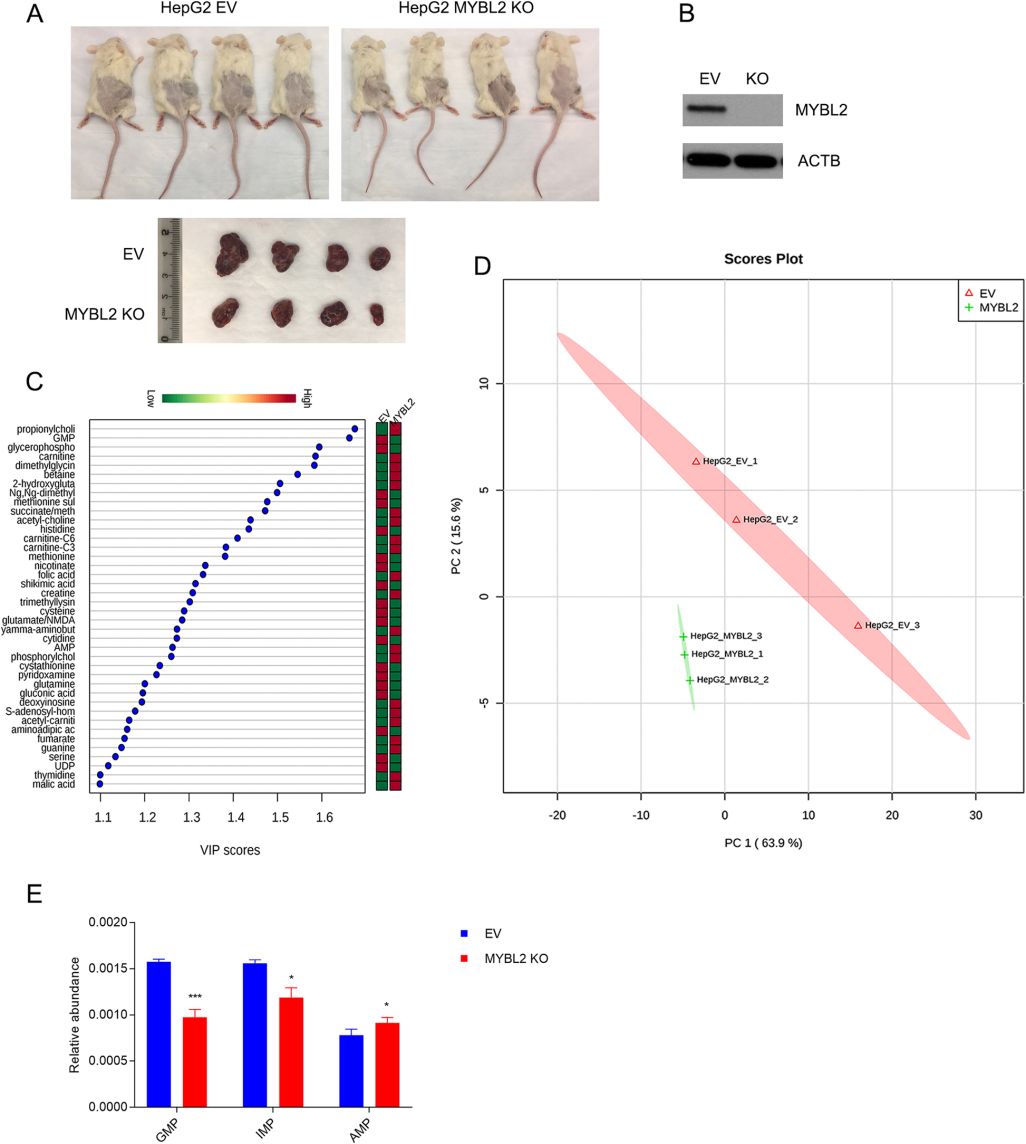
MYBL2 reprograms purine metabolism and is required for HCC tumor growth. **A** Xenograft models were established in nude mice with HepG2 cells expressing lentiCRISPRv2 empty vector (EV) or gRNA targeting MYBL2 (MYBL2 KO). Tumor sizes were decreased in tumors post MYBL2 knockout. **B** Protein abundance of MYBL2 in HepG2 cells expressing lentiCRISPRv2 empty vector (EV) or gRNA targeting MYBL2 (MYBL2 KO). **C** Metabolic profiles difference between HepG2 cells and MYBL2 knockout HepG2 cells. Variable importance in the projection (VIP) score (VIP > 1.0, metabolites with VIP > 1.2 are shown) was shown. The bar indicated relative metabolite abundance, with red representing metabolite accumulation. **D** Principal component analysis for the extracts of 3 HepG2 cells with empty vector and 3 HepG2 cells with MYBL2 knockout. No overlap was found. **E** Relative abundance of GMP, IMP and AMP in HepG2 cells expressing empty vector or CRISPR/Cas9-mediated knockout of MYBL2. *** *P* < 0.001, * *P* < 0.05

To understand the specific function of MYBL2 in HCC, we generated functional knockout of MYBL2 by CRISPR/Cas9 system in HepG2 cells, and implanted the MYBL2 knockout and parental cells in the flanks of NSG mice for 3 weeks, tumor growth were retarded after loss of MYBL2 (Fig. 4A, B). Next, we performed metabolomics analysis to see the metabolic effect of MYBL2 in HCC. Unsupervised PCA analysis showed a global change of metabolism of HepG2 cells after MYBL2 knockout (Fig. 4D). Several metabolites related to nucleotides metabolism are among the high scores, including GMP, AMP and IMP (Fig. 4C, E). VIP score of GMP was top ranked, in line with the fact that IMPDH1 is critical in reaction of IMP to XMP, a precursor of GMP. We observed a decrease of IMP and a slightly increase of AMP level after MYBL2 knockout, indicating that MYBL2 regulated synthesis of guanine nucleotide specifically via IMPDH1 (Fig. 4E).

### MYBL2 regulates de novo purine synthesis by directly activating IMPDH transcription

**Fig. 5:**
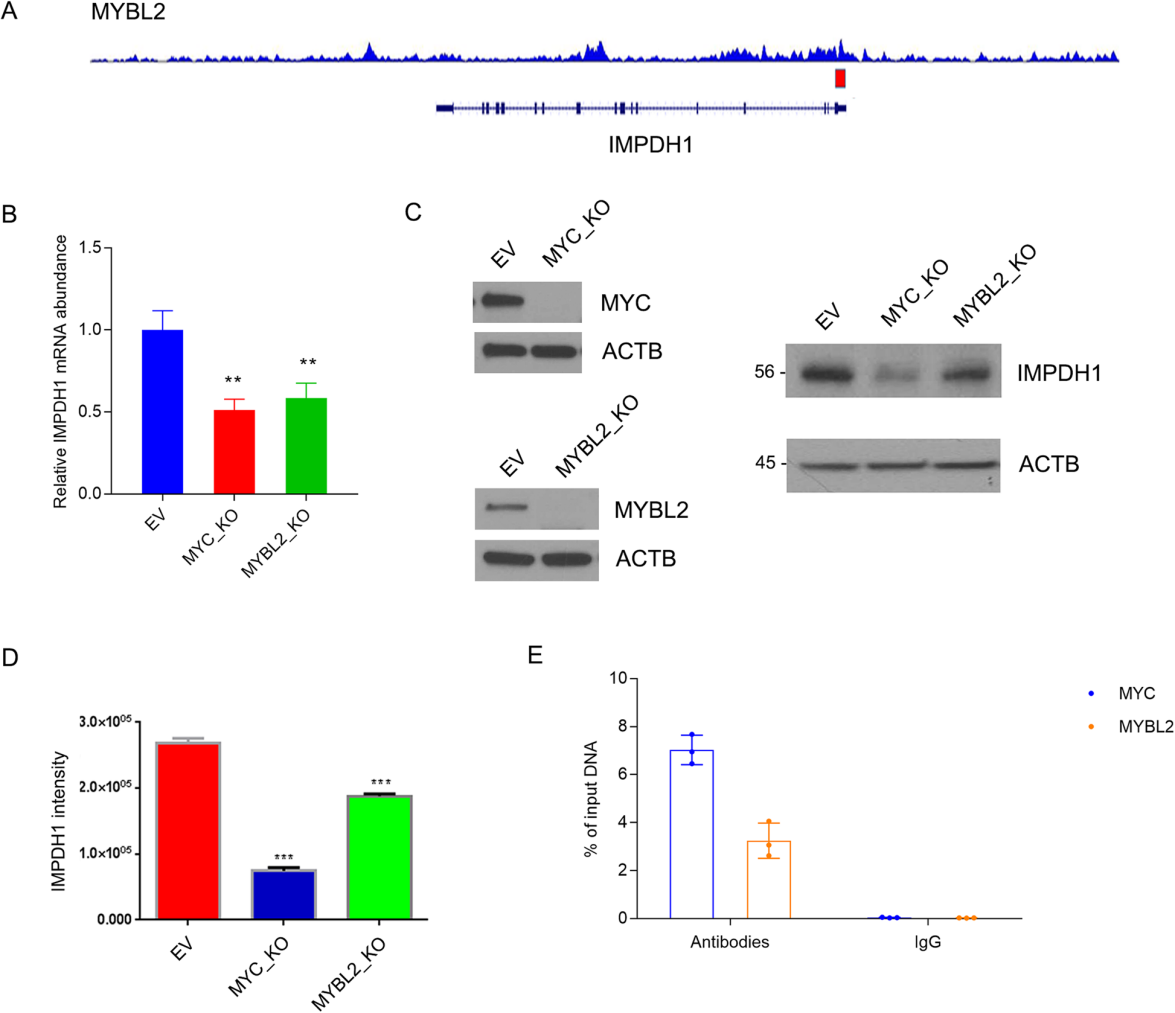
MYBL2 regulates IMPDH1 expression at transcriptional level. **A** ChIP-seq tracks showing MYBL2 binding to the promoter region of IMPDH1 in HepG2 cells. Data was from ENCODE Project. **B** mRNA abundance of IMPDH1 in HepG2 cells expressing lentiCRISPRv2 empty vector (EV) or gRNA targeting MYBL2 (MYBL2 KO) and MYC (MYC KO). ** *P* < 0.01. **C**, **D**. Protein abundance of MYC, MYBL2, and IMPDH1 in HepG2 cells in B. *** *P* < 0.001. E. qPCR for the IMPDH1 promoter after ChIP with anti-MYC, anti-MYBL2 antibodies or IgG control in HepG2 cells

**Fig. 6:**
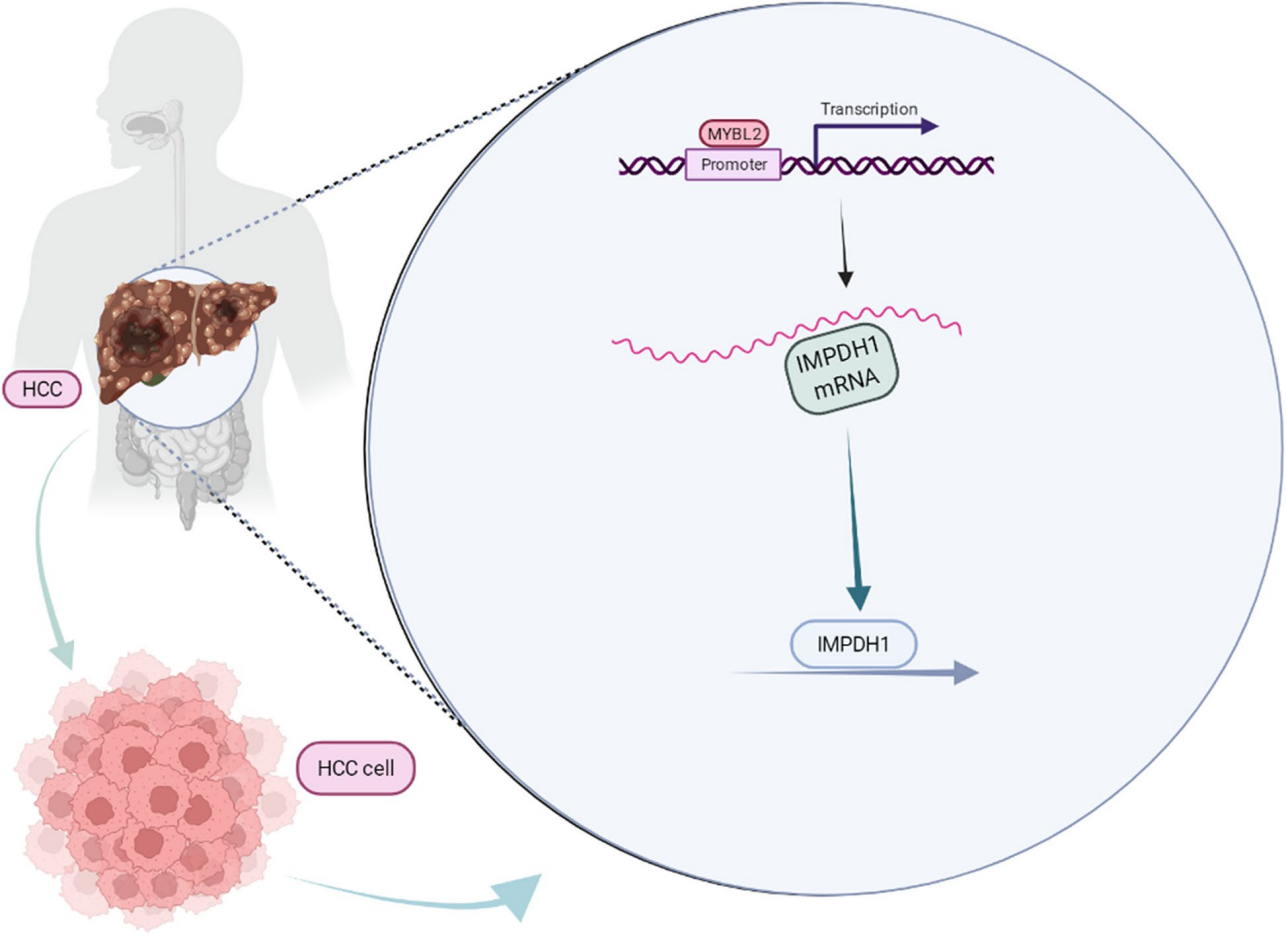
Schematic of MYBL2 regulating IMPDH1 and purine de novo synthesis. IMP, inosine monophosphate. XMP, xanthosine 5’-monophosphate

IMPDH1 is an enzyme that catalyzes the synthesis of XMP from IMP, the rate-limiting step in the de novo synthesis of guanine nucleotides. Detailed relationship between MYBL2 and IMPDH1 is not revealed yet, Chip-seq analysis from published dataset [35] showed MYBL2 could bind to the promoter region of IMPDH1 (Fig. 5A). RT-PCR showed mRNA abundance of IMPDH1 declined in HepG2 cells after MYBL2 or MYC knockout by CRISPR/Cas9 (Fig. 5B). Comparable with the effect of MYC, which is a known transcriptional regulator of IMPDH1, knocking out of MYBL2 resulted in decrease of IMPDH1 protein level (Fig. 5C), grey density reduced around 30% compared with control (Fig. 5D). To validate the direct binding of MYBL2 on the promoter region of IMPDH1, ChIP-qPCR with MYBL2 antibody was performed in HepG2 cells with MYC as a positive control. We found a good enrichment of DNA fragments in the IMPDH1 promoter region by ChIP with MYBL2, compared with ChIP with IgG control (Fig. 5E). These data suggest MYBL2 regulates de novo purine synthesis by directly activating IMPDH transcription (Fig. 6).

## Discussion

HCC remains a challenging disease worldwide due to poor prognosis and lack of treatments [36]. Oncogenic transformation like c-Myc, AKT, is considered critical events strongly associated with poor prognosis [37]. The detailed molecular mechanisms underlying HCC invasion and oncogenic transformation are still not fully understood. MYBL2 is a transcription factor, which is up-regulated during hepatocarcinogenesis and represent worse clinical outcomes [38]. Recent studies revealed MYBL2 suppression facilitates cell cycle checkpoint control and cell sentence [26], which could be regulated by microRNAs and long-non-coding RNAs [39, 40]. This seems interesting given that: (1) MYBL2 suppression is tightly involved in cell sentence and cell cycle control; (2) MYBL2 has been illustrated to promote HCC invasion; (3) proliferation and cell cycle progression needs enough nutrients for further biomass synthesis, which is more obvious in tumor cells. However, MYBL2 in regulation of cancer metabolism is rarely investigated.

Cancer is characterized by reprogramming of cellular metabolism under both direct and indirect regulation of oncogenic mutations [41]. Nucleotides are necessary for a variety of cellular processes. It has been well characterized that imbalances in nucleotide levels lead to a variety of human diseases, including cancer, immunodeficiency, aging, and a number of mitochondrial pathologies [42]. Nucleotides mainly includes purine and pyrimidine nucleotides. Purine nucleotides perform its function by constituting the building blocks for DNA and RNA, incorporating into enzyme cofactors, representing the energy source for translation and microtubule polymerization, and involving in signal transduction, angiogenesis [43] and axon guidance [7]. Purine nucleotides are generated by de novo pathways and salvage pathways, both of which are under tight regulation to balance between adenine and guanine nucleotide pools, and importantly, main an optimal energy charge along the different stages of the cell cycle [12].

IMPDH catalyzes the oxidative reaction of IMP to XMP, a rate-limiting step in guanine nucleotide biosynthesis and hence IMPDH is an essential enzyme that maintain the stability of cellular pool of guanine nucleotides. Given this, IMPDH represents an important therapeutic target that has attracted much attention due to its pluripotent functions such as the immune response [8] or cell proliferation [44]. Limited data revealed that IMPDH is highly expressed in many malignancies, and Inauzhin (INZ), a novel non-genotoxic p53 activator by inhibiting SIRT1 also inhibits IMPDH2 [45]. Though, over the last decades, IMPDH has been explored intensively for its potential as a treating target for antitumor, antiviral, antiparasitic, antibacterial and immune-suppressive activities [46]. Existed diverse group of drugs including mycophenolic acid (CellCept), mizoribine (Bredinin) and ribavirin (Virazole and Rebetol) has been widely used in clinical chemotherapy [46].

Our results were in line with limited evidence that IMPDH1 expression represent worse clinical outcomes, while combining MYBL2 and IMPDH1 together, prognosis gap is larger, indicting they were important in HCC progression, and there is a synergy between the two. Correlation analysis showed MYBL2 was positively associated with IMPDH1. Chip-seq showed MYBL2 is a transcription factor for IMPDH1, so it might regulate IMPDH1 at transcriptional level, which linked MYBL2 to purine anabolism through key enzyme control. RT-PCR validated suppressed MYBL2 impaired IMPDH1 transcriptional level, in line with the prediction.

While CRISPR/Cas9 is a new tool for gene editing, its application in metabolomics made metabolic reprogramming alteration clearer, especially critical molecular in regulation of cancer metabolism. In this study, MYBL2 is knocked out by the same method, alteration of metabolic profiles revealed MYBL2 positively correlated with GMP, while in process of GMP generation, and IMPDH1 is essential and a rate-limiting enzyme. This also illustrated the regulation of MYBL2 on IMPDH1 in HCC cells. GMP is the precursor of GDP and GTP, which is an energy mole particularly important for protein synthesis in rapidly dividing cells, such as tumor cells [47, 48]. So, MYBL2 regulated purine anabolism, which meant it was also in control of GTP, influencing beyond biomasses synthesis, of great important in cancer cell proliferation.

IMPDH1 is an enzyme catalyzes a crucial step in de novo purine synthesis, especially for GMP. Knocking out of MYBL2 in HCC cells by CRISPR/Cas9, which made a dramatic alteration in nucleotides metabolic profiles in HCC, especially in purine anabolism as described in our study. Tumor bearing models by HCC cells also exhibited suppression in purine anabolism with MYBL2 knocking out. Together, these results illustrated guanine synthesis is controlled by MYBL2 in both vivo and vitro, thus influenced proliferation in HCC cells.

Here in our study, we first demonstrated IMPDH1 expression is regulated by transcription factor MYBL2, and thus linking oncogenic transformation and purine catabolism together. Of note, this is the first study illustrated MYBL2 regulated purine anabolism and contributed to HCC tumorigenesis. The study also provides evidence that MYBL2 is a promising target for cancer treatments and proposed a possible mechanism of HCC progression.

## Supplementary Information

**Additional file 1.**

**Additional file 2.**

**Additional file 3.**

**Additional file 4.**

**Additional file 5.**

## Data Availability

The datasets generated and/or analyzed during the current study are available in the TCGA pan-caner repository (https://portal.gdc.cancer.gov). Further inquiries can be directed to the corresponding author.

## Abbreviations

HCC: Hepatocellular Carcinoma
Chip-seq: Chromatin immunoprecipitation sequence
ChIP-qPCR: Chromatin immunoprecipitation quantitative polymerase chain reaction
CCA: Cholangiocarcinoma
HBV: Hepatitis B virus
HCV: Hepatitis C virus
NAFLD: Nonalcoholic fatty liver disease
PRPP: Phosphoribosyl pyrophosphate
IMPDH: IMP dehydrogenase
XMP: Xanthosine 5’-monophosphate
MTX: Methotrexate
MYBL2: Homolog-like2 B-Myb
IHC: Immunohistochemical
INZ: Inauzhin

## Declarations

All experimental protocols were approved by Animal Experimentation Ethics Committee of Union Hospital, Tongji Medical College, Huazhong University of Science and Technology. All methods were carried out in accordance with relevant guidelines and regulations and are reported in accordance with ARRIVE guidelines for the reporting of animal experiments.
