# Supplementary figures and images for: MYBL2 regulates de novo purine synthesis by transcriptionally activating IMPDH1 in hepatocellular carcinoma cells
Source: BMC Cancer. 2022 Dec 9;22:1290. doi: 10.1186/s12885-022-10354-4 (PMC9733023; doi:10.1186/s12885-022-10354-4)

Figure 4B

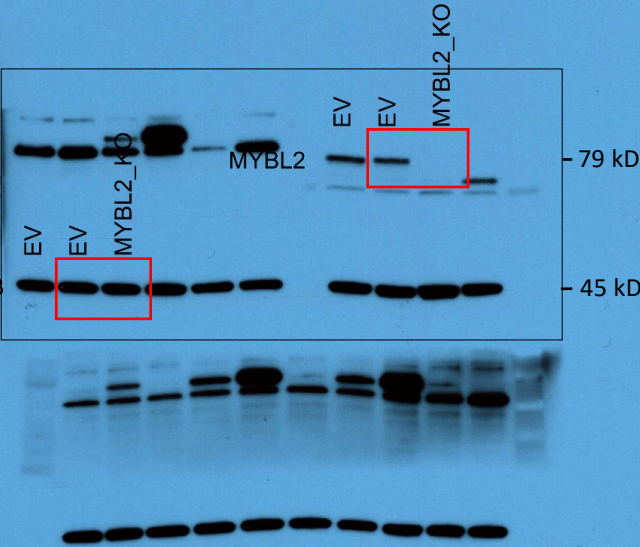

Supplement: Supplementary file 1 — Additional file 1. [file 12885_2022_10354_MOESM1_ESM.pdf]

Figure 5C

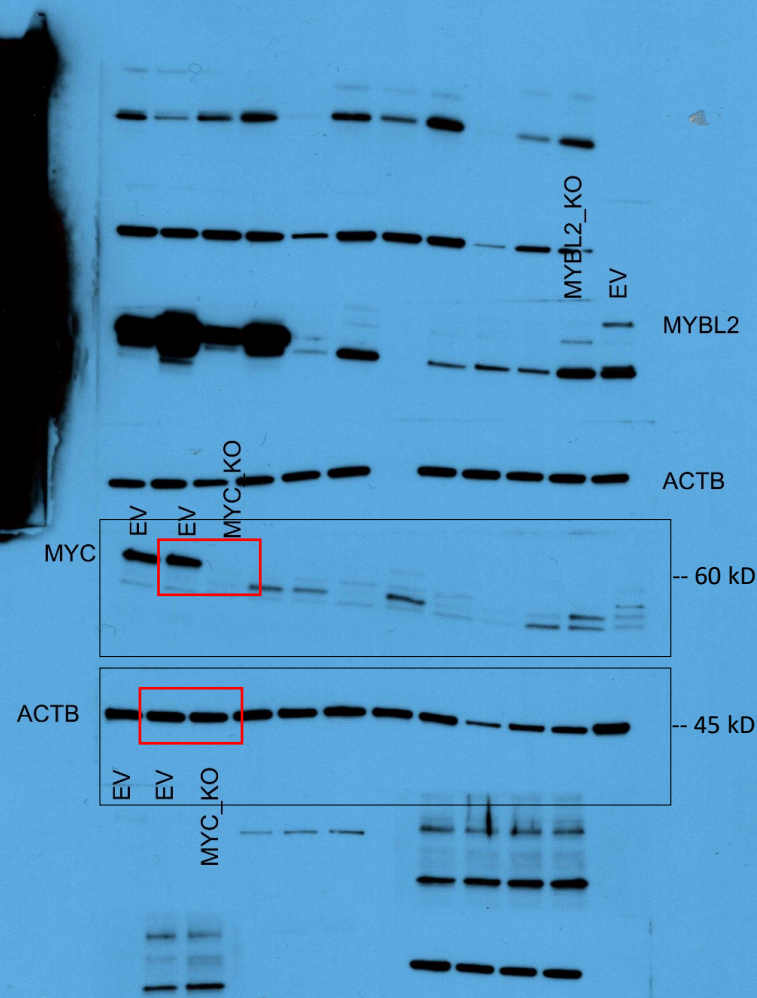

Supplement: Supplementary file 2 — Additional file 2. [file 12885_2022_10354_MOESM2_ESM.pdf]

Figure 5C

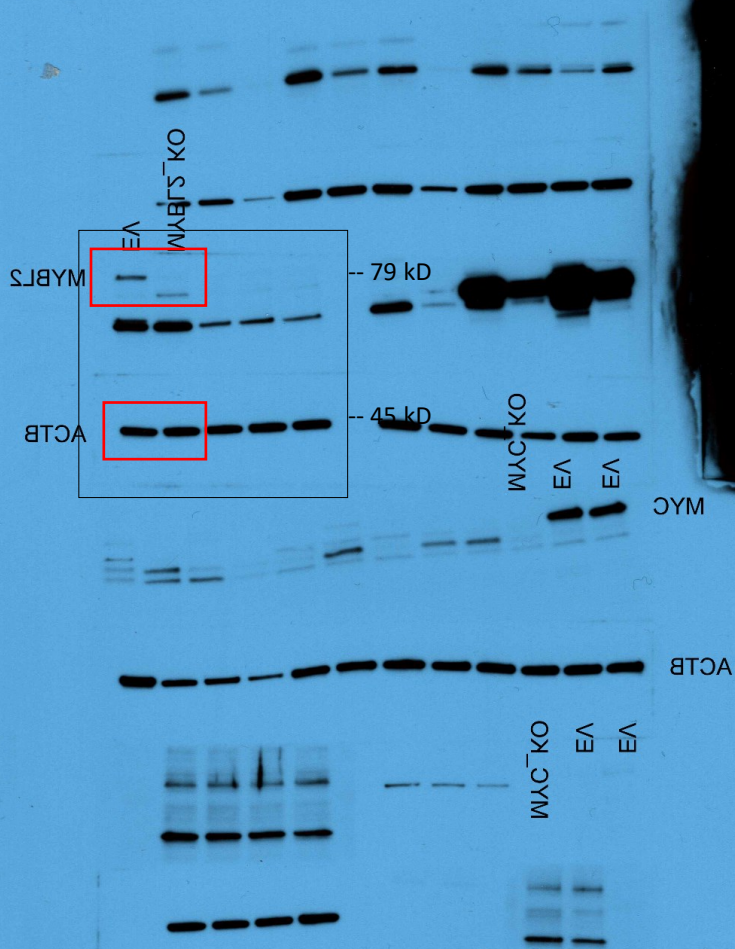

Supplement: Supplementary file 3 — Additional file 3. [file 12885_2022_10354_MOESM3_ESM.pdf]

Figure 5C

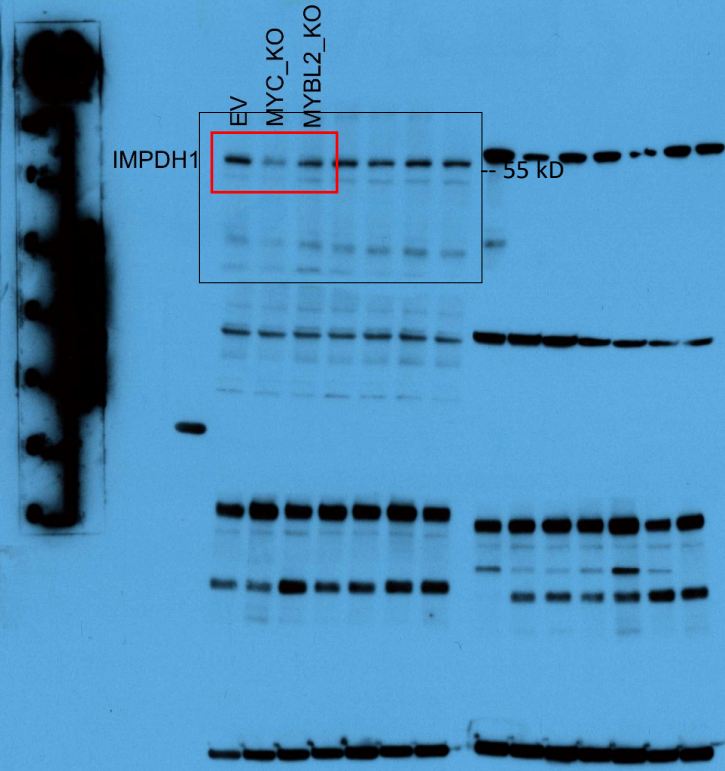

Supplement: Supplementary file 4 — Additional file 4. [file 12885_2022_10354_MOESM4_ESM.pdf]

Figure 5C

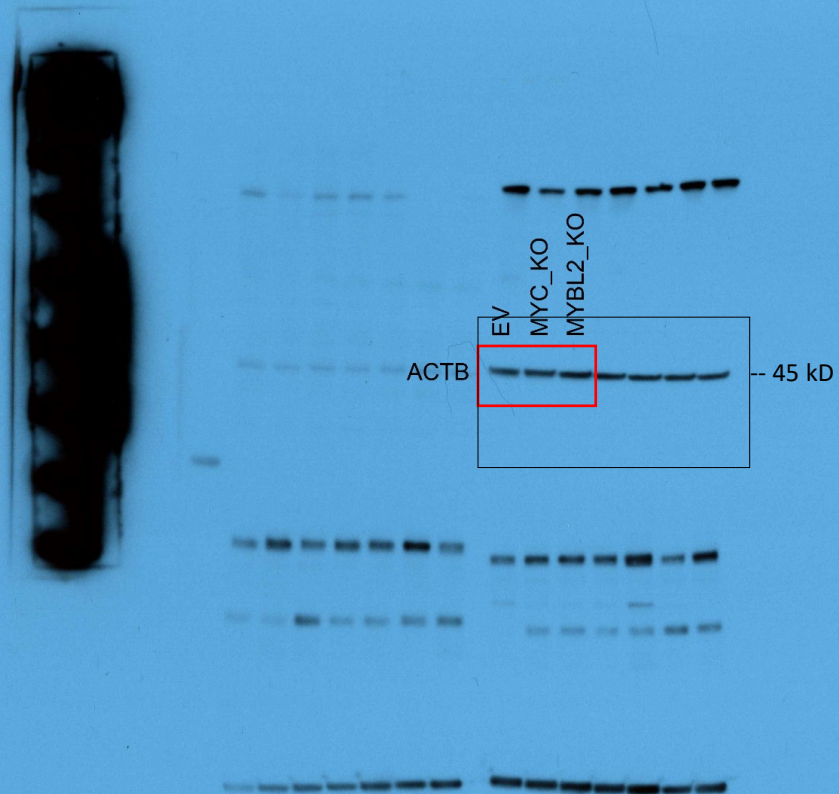

Supplement: Supplementary file 5 — Additional file 5. [file 12885_2022_10354_MOESM5_ESM.pdf]
